# Decision Fatigue in the Attending Surgeon: The Neural Accumulation of Micro-decisions Across an Operating Day

**DOI:** 10.7759/cureus.110327

**Published:** 2026-06-05

**Authors:** Derick Rodriguez-Reyes, Joseph Salem-Hernández, Ivelisse Pedreira-García, Roberto Cardona-Quiñones, Saidy Salem-Hernández, Norman Ramírez

**Affiliations:** 1 Department of Orthopedic Surgery, Ponce Health Sciences University, Ponce, PRI; 2 Department of Clinical Psychology, University of Puerto Rico at Río Piedras, San Juan, PRI; 3 Department of Neurology, Veterans Affairs Boston Healthcare System, Boston, USA

**Keywords:** cognitive load, decision fatigue, micro-decisions, neural mechanisms, prefrontal cortex, surgeon performance, surgical outcomes

## Abstract

Decision fatigue represents a progressive deterioration in decision-making quality arising from the cumulative cognitive burden of repeated choices. Attending surgeons navigate hundreds of micro-decisions throughout an operating day, encompassing instrument selection, tissue handling, and real-time adjustments in surgical approach, particularly when unexpected intraoperative findings demand immediate adaptive reasoning, each drawing upon finite neural resources. This review synthesizes current evidence on the neural mechanisms underlying decision fatigue in surgeons, with particular emphasis on prefrontal cortex (PFC) depletion, neurotransmitter dynamics, and metabolic constraints. We propose a taxonomy of intraoperative micro-decisions organized by strategic versus tactical, perceptual versus conceptual, time-critical versus deliberative, and reversible versus irreversible dimensions. Evidence demonstrates that decision fatigue manifests in measurable performance decrements, including prolonged operative times, increased error rates, and elevated complication risks, particularly in later cases of the operating day when unexpected intraoperative findings compound the risk posed by accumulated cognitive depletion. Individual factors such as experience, stress resilience, and sleep quality, together with systemic variables including case scheduling, team dynamics, and technology support, moderate these effects. Mitigation strategies encompass individual-level interventions (strategic breaks, nutrition optimization, cognitive training), team-based approaches including a second attending surgeon as cognitive monitor, technological cognitive offloading, and system-level organizational change. Critical research gaps include the need for real-time intraoperative neural monitoring, validated micro-decision taxonomies, and prospective trials of mitigation protocols. Understanding decision fatigue as a neurobiological phenomenon rather than a character flaw is essential for developing evidence-based strategies that optimize surgeon performance and patient safety.

## Introduction and background

The modern attending surgeon operates within an environment of relentless decision-making. From the moment of surgical incision to final closure, surgeons execute a continuous stream of choices, both deliberate and strategic as well as rapid and intuitive, that collectively determine procedural success and patient safety. While individual high-stakes decisions receive considerable attention in surgical education and quality-improvement initiatives, the cumulative cognitive burden of hundreds of micro-decisions throughout an operating day remains poorly characterized.

Decision fatigue, a concept extensively studied in psychology and behavioral economics, refers to the deteriorating quality of decisions made after a prolonged session of decision-making [1]. It is distinct from, though related to, physical fatigue, mental fatigue broadly construed, and cognitive overload. Physical fatigue manifests as motor and muscular impairment with direct perceptual correlates. Mental fatigue is a generalized depletion of cognitive resources arising from sustained cognitive effort across domains. Cognitive overload represents acute exceedance of available processing capacity, typically triggered by a specific task or environmental demand. Decision fatigue, by contrast, is a cumulative phenomenon specific to the quality of successive decisions: it operates insidiously, degrading judgment, impulse control, and the capacity for complex reasoning while the decision-maker often remains subjectively unaware of the deficit [2]. In surgical practice, this phenomenon takes on particular significance: surgeons frequently perform multiple complex procedures sequentially, with each case demanding sustained attention, technical precision, and adaptive problem-solving under conditions of uncertainty and time pressure [3].

The epidemiological context underscores the urgency of this issue. Surgeon burnout and fatigue are prevalent across specialties, with surveys consistently reporting that a substantial proportion of surgeons operate while experiencing significant cognitive fatigue. The consequences extend beyond the operating room, with mental workload profiles linked to presenteeism, absenteeism, and diminished occupational performance [4]. Despite this recognition, the specific mechanism of micro-decision accumulation, distinct from general fatigue, has not been adequately characterized in the surgical fatigue literature, which has focused predominantly on physical fatigue, duty-hour effects, and gross performance metrics.

The neural substrate of decision-making resides primarily in the prefrontal cortex (PFC), a brain region responsible for executive functions including working memory, cognitive flexibility, inhibitory control, and goal-directed behavior. Functional near-infrared spectroscopy (fNIRS) studies have revealed that the PFC exhibits measurable changes in activation patterns during sustained cognitive work, with direct implications for performance stability. Understanding decision fatigue through a neurobiological lens, as a consequence of finite neural resources rather than a moral failing or lack of dedication, is essential for developing rational, evidence-based interventions aimed at protecting both the surgeon's well-being and patient safety. The present review synthesizes current evidence across neural mechanisms, micro-decision taxonomy, temporal dynamics, performance outcomes, moderating factors, mitigation strategies, and future research directions.

## Review

Methods

This manuscript is a narrative review. Literature was identified through systematic searches of PubMed, MEDLINE, and Google Scholar, covering publications from 2000 through 2025. Key search terms included: decision fatigue, surgeon cognitive load, prefrontal cortex surgery, intraoperative fatigue, surgical performance fatigue, cognitive load theory surgery, fNIRS surgeons, and sleep deprivation surgeon performance. Reference lists of included articles were manually screened to identify additional relevant sources. Inclusion criteria encompassed peer-reviewed studies in English addressing decision fatigue, cognitive load, or fatigue effects in surgical or high-stakes decision-making contexts, as well as mechanistic neuroscience literature directly relevant to prefrontal cortex function and executive decision-making. As a narrative review, this manuscript does not employ systematic review or Preferred Reporting Items for Systematic Reviews and Meta-Analyses (PRISMA) methodology; formal risk-of-bias assessment for individual studies was not performed, and readers should interpret mechanistic claims with the understanding that supporting studies vary in design rigor, sample size, and generalizability. Thirteen primary references ranging from 2008 to 2025 are cited, all directly relevant to the topic of decision fatigue in the attending surgeon.

Neural mechanisms of decision fatigue

**Figure 1:**
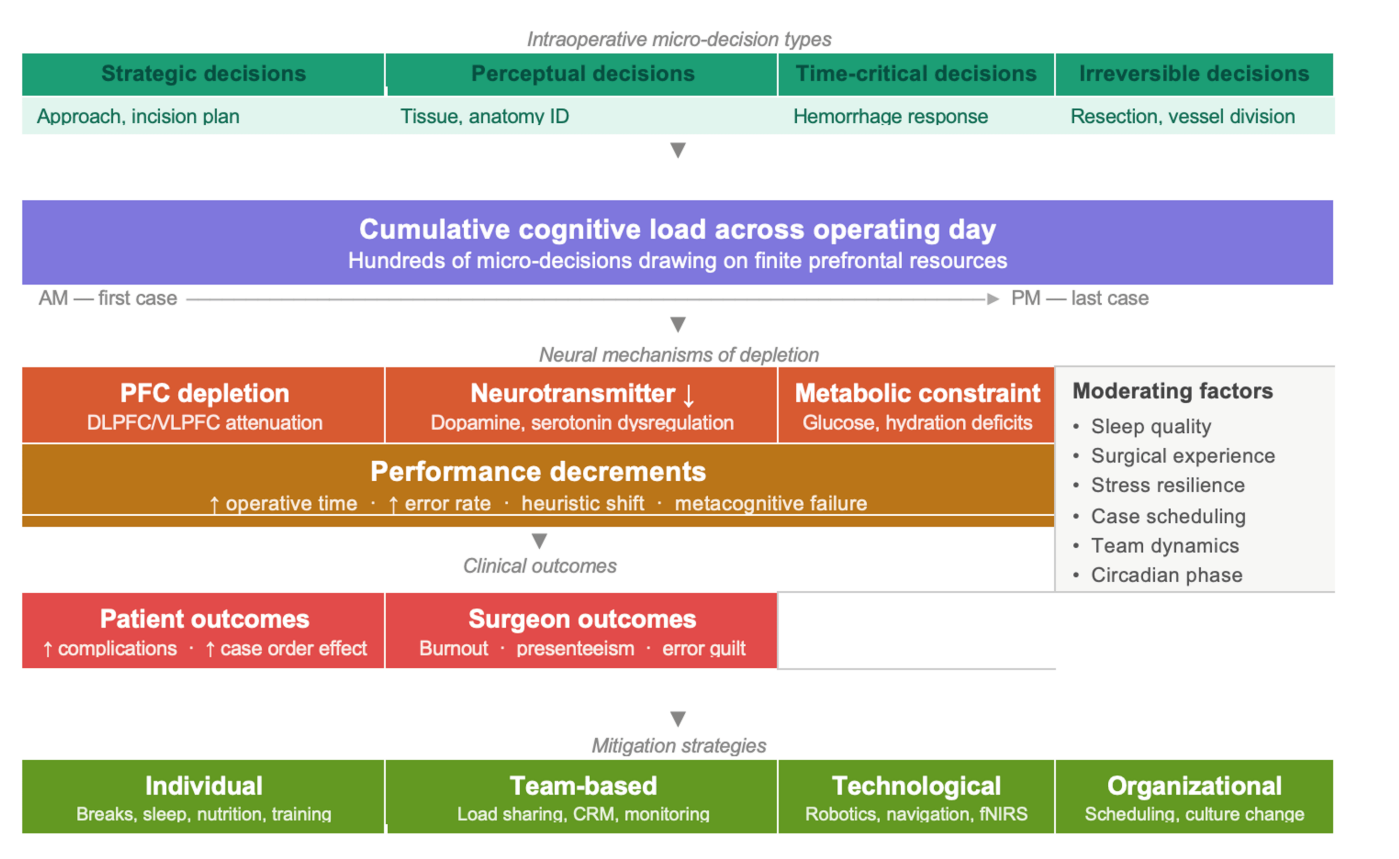
Decision fatigue in the attending surgeon: conceptual model from micro-decision accumulation to clinical outcomes. The figure depicts the proposed conceptual pathway by which micro-decision accumulation across an operating day leads to progressive prefrontal cortex (PFC) depletion through three interacting mechanisms: PFC attenuation (as measured by fNIRS), neurotransmitter dysregulation (dopaminergic and serotonergic), and metabolic constraint (cerebral glucose and hydration deficits). These neural mechanisms converge to produce measurable performance decrements, including prolonged operative times, increased error rates, a shift from controlled to heuristic-based processing, and metacognitive failure, which in turn manifest as adverse patient outcomes (increased complications, case order effect) and surgeon occupational outcomes (burnout, presenteeism, error guilt). Moderating factors (right panel) include sleep quality, surgical experience, stress resilience, case scheduling, team dynamics, and circadian phase. The bottom tier depicts the four levels of mitigation strategy: individual, team-based, technological, and organizational. The dashed feedback arrow indicates that successful mitigation interrupts the accumulation-to-outcome pathway. Figure was created entirely using Microsoft Word (Microsoft Corporation, Redmond, Washington, USA), Apple Freeform (Apple Inc., Cupertino, CA, USA), and SciSpace (PubGenius Inc.; https://scispace.com). CRM: crew resource management; DLPFC: dorsolateral prefrontal cortex; fNIRS: functional near-infrared spectroscopy; PFC: prefrontal cortex; VLPFC: ventrolateral prefrontal cortex.

The proposed conceptual model linking micro-decision accumulation to clinical outcomes, across neural mechanisms, performance decrements, and mitigation strategies, is illustrated in Figure 1.

Prefrontal cortex depletion

The dorsolateral (DLPFC) and ventrolateral (VLPFC) regions of the PFC serve as the primary neural substrate for executive decision-making, working memory maintenance, and cognitive control. fNIRS studies of surgical residents performing laparoscopic tasks under time pressure, where time pressure in real operative settings is predominantly event-driven, generated by unexpected complications, sudden hemorrhage, or unanticipated anatomy rather than clock-watching alone, have revealed distinct patterns of PFC activation associated with performance stability versus decline [3]. Residents demonstrating stable performance under temporal stress exhibited sustained increases in oxygenated hemoglobin concentration across bilateral VLPFC and right DLPFC, indicative of preserved attention and concentration; in contrast, residents whose performance deteriorated showed PFC deactivation, potentially representing cognitive overload or task disengagement [3]. These findings align with the broader neuroscience literature demonstrating that the PFC is particularly vulnerable to depletion during sustained cognitive effort. The phenomenon of "losing your nerve," wherein surgeons experience performance degradation under temporal demands, has been directly linked to prefrontal attenuation measured via fNIRS; Modi et al. demonstrated that junior residents showed PFC deactivation and technical decline under time pressure, while senior residents maintained prefrontal engagement and performance stability, confirming that decision fatigue is a measurable neurophysiological state.

As cognitive load accumulates throughout an operating day, the PFC's capacity to maintain higher-order functions such as error monitoring, conflict resolution, and adaptive strategy selection progressively diminishes, leading to a cascade of performance decrements [5]. Surgeons may shift from controlled, deliberate processing to more automatic, heuristic-based decision-making, a transition that, while cognitively economical, increases vulnerability to systematic errors and biases [1]. Critically, PFC function is modulated by sleep quality, circadian rhythms, and acute stressors. Sleep deprivation, common among surgeons, produces PFC dysfunction with documented impairments in attention, working memory, and decision-making; meta-analytic data indicate that sleep loss in physicians consistently degrades cognitive and clinical task performance in a dose-dependent manner. The interaction between chronic sleep debt and acute intraoperative cognitive demands creates a particularly high-risk scenario for decision fatigue. Evidence supports targeting seven to nine hours of consolidated sleep per night; fragmented or curtailed sleep, common in surgical culture due to call schedules and early operative starts, fails to provide the restorative neural consolidation necessary for full cognitive recovery.

Dopaminergic and serotonergic pathways

Neurotransmitter systems, particularly dopamine and serotonin, play critical roles in modulating PFC function and decision-making processes. The dopaminergic system operates on an inverted-U function: both insufficient and excessive dopamine may impair PFC-dependent cognition, with optimal performance thought to occur at intermediate levels [3]. During sustained decision-making, dopamine depletion may occur through repeated activation of reward-evaluation circuits without adequate recovery, potentially manifesting as reduced motivation, impaired reward sensitivity, and a shift toward risk-averse or default choices [1]. In the surgical context, this could translate to reluctance to pursue technically demanding but clinically optimal approaches in favor of safer, more routine alternatives, a phenomenon possibly contributing to the case order effect wherein later surgical cases exhibit more conservative decision-making patterns [6].

Serotonin, widely distributed throughout the cortex, modulates mood, impulse control, and long-term planning. Mental fatigue may disrupt serotonergic signaling, contributing to the erosion of inhibitory control observed in fatigued surgeons, manifesting clinically as increased susceptibility to cognitive biases, reduced patience with complex problem-solving, and heightened emotional reactivity to intraoperative stressors [5]. The interaction between dopaminergic and serotonergic systems is complex and context-dependent; stress-induced release of glucocorticoids and noradrenaline can acutely enhance PFC function but, when sustained, may lead to receptor downregulation and functional impairment. The temporal dynamics of these neurochemical changes over the course of an operating day remain poorly characterized and likely contribute to the progressive nature of decision fatigue. However, direct mechanistic evidence in surgical contexts remains inferential [4].

Glucose metabolism and energy depletion

The brain consumes approximately 20% of the body's glucose and oxygen at rest, with the PFC being particularly metabolically demanding. Cognitive tasks requiring executive function produce measurable increases in regional cerebral glucose metabolism. The glucose depletion hypothesis of decision fatigue posits that sustained cognitive effort may deplete available glucose in brain regions supporting executive function, leading to performance decrements; evidence suggests that glucose availability can influence cognitive performance, particularly during demanding tasks, although effects are modest and context-dependent [1]. In the surgical context, surgeons often work through meals, experiencing prolonged fasting that may exacerbate metabolic constraints on cognitive function. Dehydration, common during long procedures, may further impair cognitive performance through reduced cerebral blood flow and altered neurotransmitter function. The combination of high metabolic demand, limited substrate availability, and physiological stress creates conditions conducive to cognitive depletion [7].

Micro-decision taxonomy in surgery

The following taxonomy is proposed as a conceptual framework to organize future empirical research into intraoperative decision-making. It is not an empirically validated classification system; prospective observational studies across diverse surgical specialties and procedure types will be necessary to test, refine, and validate these dimensions.

Types of intraoperative decisions

Surgical procedures comprise a heterogeneous array of decisions varying in complexity, time pressure, reversibility, and consequence. Strategic decisions involve high-level planning and approach selection, such as choice of incision, sequence of dissection, and decision to proceed with or abort a resection, and typically occur at discrete timepoints requiring explicit deliberation with significant consequences for overall procedural success. In contrast, tactical decisions are the moment-to-moment choices that implement strategic plans, such as instrument selection, angle of approach, and timing of hemostatic interventions; while individually minor, they occur at high frequency and collectively determine technical execution quality [2].

A further distinction exists between perceptual decisions, which rely primarily on sensory input and pattern recognition, such as identifying anatomical structures and assessing tissue quality, and conceptual decisions, which require abstract reasoning and integration of multiple information sources. Conceptual decisions place greater demands on working memory and executive function, making them particularly vulnerable to fatigue effects [3]. Time-critical decisions must be made within seconds in response to dynamic intraoperative events, such as sudden hemorrhage or anesthetic complications, relying heavily on pattern recognition and procedural memory. Deliberative decisions allow more extended consideration, such as whether to extend a resection margin or how to manage an unexpected anatomical variant; while less immediately stressful, they may be more susceptible to fatigue-induced degradation because they require sustained executive function [5].

Finally, the reversibility dimension influences decision-making strategy: easily adjustable choices differ fundamentally from irreversible ones, such as division of critical structures or extent of resection, with the latter prompting higher subjective stress and more careful deliberation that places additional demands on depleted neural resources [8]. The convergence of accumulated cognitive depletion with the unpredictability of intraoperative surprises, unexpected anatomical variants, occult bleeding, and sudden hemodynamic instability creates a compounded risk that is distinct from the aggregate outcome data captured by the case order effect and represents a strong argument for scheduling complex or high-uncertainty cases earlier in the operating day.

Cognitive load categorization

Cognitive load theory provides a useful framework for categorizing surgical micro-decisions. Intrinsic load reflects the inherent complexity of the decision, determined by the number of elements that must be simultaneously considered and their interrelationships; deciding whether to proceed with complex vascular reconstruction in the setting of hostile anatomy, poor tissue quality, and hemodynamic instability exemplifies high intrinsic load [8]. Extraneous load arises from factors peripheral to the core decision, including poor visualization, equipment malfunction, and communication difficulties, representing wasted cognitive capacity that depletes finite resources without contributing to decision quality. Minimizing extraneous load through optimal operating room design, equipment reliability, and structured team communication protocols is therefore a key modifiable target for intervention [5]. Germane load, directed toward learning and schema construction, is productive in the long term but still contributes to acute cognitive fatigue. When total load approaches or exceeds available cognitive capacity, performance degrades, and the cumulative effect of sustained high load across an operating day produces progressive depletion of neural resources, manifesting as decision fatigue [8].

Temporal dynamics of decision accumulation across an operating day

The temporal pattern of decision fatigue accumulation in surgeons exhibits both predictable and variable features. A well-documented "case order effect" shows that surgical performance and outcomes deteriorate across sequential cases performed by the same surgeon on the same day. Bagrodia et al. examined 4,471 robotic-assisted radical prostatectomies and found that later cases were associated with significantly longer operative times and higher complication rates compared to earlier cases, even after controlling for case complexity and patient factors, suggesting progressive cognitive depletion rather than simple patient selection effects [5]. Studies of laparoscopic cholecystectomy and other common procedures have similarly demonstrated increased error rates and prolonged operative times in afternoon versus morning cases [1].

The temporal dynamics of cognitive load accumulation appear to follow a nonlinear pattern. Initial cases of the day may benefit from a "warm-up" effect, with optimal performance occurring in the second or third case after the surgeon achieves full engagement but before significant fatigue has accumulated; subsequently, performance gradually declines with a rate that may accelerate in later cases as compensatory mechanisms become exhausted [6]. The Flush Model proposed by Laulan et al. conceptualizes mental fatigue as accumulating like water in a container, with complex cases adding more "volume" and potentially causing overflow if not adequately managed [8]. Several modulating factors shape this trajectory: longer and more complex cases impose greater cognitive demands and produce more rapid fatigue accumulation; inter-case recovery intervals provide partial cognitive restoration, with even brief breaks demonstrating measurable benefit in randomized trials [5,7]. Cognitive performance also exhibits circadian rhythmicity, with most individuals showing peak performance in late morning and early afternoon and nadirs in the early morning and late evening, a pattern compounded in surgeons operating outside their optimal circadian phase during overnight call [9].

Chronic sleep restriction, common across surgical specialties, produces cumulative cognitive deficits that interact synergistically with acute decision fatigue; McCormick et al. used actigraphy to demonstrate that a substantial proportion of surgical residents' operating time was spent at less than 80% mental effectiveness [9]. Frequent interruptions and competing demands, communicating with team members, teaching trainees, and responding to pages, fragment attention and further increase cognitive load throughout the operating day [5].

Impact on surgical performance and patient outcomes

Technical Performance Metrics

Decision fatigue manifests in measurable decrements across multiple dimensions of surgical performance with downstream effects on patient outcomes. Multiple studies have documented prolonged operative times in later cases of the day, even after controlling for case complexity, and increased operative time itself carries independent risks, including greater anesthesia exposure and elevated infection risk [6]. Systematic reviews of mental fatigue effects on surgical performance consistently demonstrate increased error rates and degraded technical skills in fatigued surgeons. Kahol et al. found that fatigue produced significant impairments in both psychomotor and cognitive skills comparable in magnitude to the effects of alcohol intoxication [10]. Gerdes et al. similarly demonstrated that fatigue impaired cognitive and psychomotor performance in both surgical residents and attending surgeons, with attending surgeons showing a preserved but not immune response, experience attenuates but does not eliminate the effects of fatigue [11].

Clinical Judgment Impairment

Beyond technical metrics, decision fatigue specifically impairs the quality of clinical judgments. Degraded clinical judgment is not an abstract construct; it manifests as choosing a technically easier but suboptimal approach, failing to recognize an early sign of a complication, misidentifying an anatomical structure, or erroneously assessing that tissue quality is adequate to proceed, decisions that can directly harm patients. Persson et al. analyzed Swedish registry data and found evidence of decision fatigue effects on surgeons' clinical decision-making with implications for treatment selection and resource utilization [1]. Fatigued surgeons may exhibit increased reliance on heuristics and default options, reduced consideration of alternative approaches, and impaired integration of complex information [2].

An important and clinically consequential finding across studies is that surgeons' subjective perception of their own fatigue and performance impairment frequently underestimates objective deficits. Schlosser et al. found that surgeons regularly rated their performance as adequate even when objective measures demonstrated significant impairment, a metacognitive failure that represents a major barrier to voluntary self-regulation [12]. This underscores the need for objective monitoring and system-level safeguards rather than sole reliance on individual self-assessment. Mental workload profile has also been linked to occupational outcomes, including presenteeism and absenteeism, suggesting that the consequences of unmanaged decision fatigue extend beyond the immediate operating encounter [4].

Individual and systemic moderating factors

Susceptibility to and manifestation of decision fatigue vary substantially across individual surgeons and surgical systems. At the individual level, stress resilience constitutes a key moderating factor: Modi et al. identified distinct neural signatures in stress-resilient surgeons, who maintained sustained prefrontal activation under time pressure, versus vulnerable surgeons who showed prefrontal deactivation and performance decrements under identical conditions [3]. Sleep quality and quantity represent another foundational individual moderator. Chronic sleep restriction produces cumulative cognitive deficits that interact synergistically with acute decision fatigue; a meta-analysis by Philibert examining 60 studies found that sleep loss in resident physicians produced consistent impairments in cognitive performance, vigilance, and clinical task execution across both acute and chronic deprivation conditions. Physical fitness, chronic health status, and experience level further modulate susceptibility: expert surgeons process many technical maneuvers with minimal conscious attention, preserving cognitive resources for higher-level decisions, though expertise does not eliminate the risk of fatigue effects [11].

At the systemic level, case scheduling and sequencing substantially influence fatigue accumulation: scheduling highly complex cases late in the day, when decision fatigue is maximal, represents a modifiable risk factor amenable to intelligent sequencing interventions [6]. Effective surgical teams can distribute cognitive load across multiple individuals; experienced assistants, well-trained nurses, and engaged anesthesiologists can anticipate needs, identify potential problems, and offer decision support, effectively serving as cognitive offloading resources [5]. Conversely, dysfunctional team dynamics, poor communication, and role ambiguity increase cognitive load and exacerbate fatigue effects. Organizational culture surrounding fatigue significantly influences individual behavior: in cultures that valorize endurance and stigmatize acknowledgment of fatigue, particularly given the professional-reputational consequences of complications attributable to cognitive impairment, surgeons are less likely to request breaks, delegate decisions, or decline cases when cognitively depleted [3]. This stigma is precisely what perpetuates unsafe practice patterns and demands system-level cultural change. Regulatory and economic pressures, including productivity expectations and absence of work-hour limits for attending surgeons in many jurisdictions, create systemic pressures that amplify fatigue risk and require system-level rather than purely individual solutions [9].

Parallels with high-reliability industries are instructive. Aviation's crew resource management (CRM) framework has demonstrated that systematic acknowledgment of human cognitive limits, combined with standardized protocols for team communication and fatigue mitigation, substantially reduces error rates. Emergency medicine and anesthesiology have developed analogous fatigue management frameworks with measurable patient safety benefits. These models offer directly translatable principles for surgical practice, including the normalization of fatigue acknowledgment, structured handoffs, and mandatory rest periods after cognitively demanding work.

Mitigation strategies and cognitive offloading approaches

Addressing decision fatigue requires a multifaceted approach spanning individual, team, technological, and organizational levels. The evidence base supporting specific interventions varies considerably in rigor and surgical-context specificity; Table 1 summarizes the key mitigation strategies organized by level with proposed mechanisms and evidence grades.

**Table 1:** Mitigation strategies for decision fatigue in the attending surgeon, organized by intervention level. Evidence grades: RCT: randomized controlled trial; meta-analysis: pooled analysis of multiple studies; observational: cohort, registry, or prospective non-randomized study; prospective controlled: non-randomized prospective comparative study; conceptual: mechanistically grounded but without direct empirical validation in surgical settings; expert consensus: narrative review or expert opinion; future direction: proposed but not yet empirically tested; AM: ante meridiem; HPA: hypothalamic-pituitary-adrenal; PFC: prefrontal cortex; fNIRS: functional near-infrared spectroscopy. Numbers in brackets correspond to the manuscript reference list.

Level	Intervention	Proposed mechanism	Evidence	Key reference(s)
Individual	Scheduled intraoperative and inter-case breaks	Partial PFC recovery; reduction of acute metabolic and neurotransmitter depletion; measurable fatigue reduction on validated scales	RCT	Engelmann et al., 2011 [7]
Individual	Sleep optimization (7-9 h per night; strategic naps on call)	Restoration of PFC function; reversal of chronic sleep-debt-driven cognitive deficits; dose-dependent improvement in vigilance and clinical task performance	Meta-analysis	Philibert (cited in text); McCormick et al., 2012 [9]
Individual	Nutritional optimization and hydration maintenance	Maintenance of cerebral glucose availability; prevention of dehydration-related reduction in cerebral blood flow and neurotransmitter synthesis	Conceptual	Persson et al., 2019 [1]
Individual	Mindfulness and cognitive resilience training	Reduced stress reactivity; attenuated HPA-axis response; enhanced prefrontal stress-resilience neural signatures on fNIRS	Observational	Modi et al., 2019 [3]
Individual	Mental rehearsal of surgical procedures	Schema consolidation; reduced intrinsic cognitive load for familiar sequences; preserves PFC resources for novel intraoperative decisions	Observational	Modi et al., 2019 [3]
Team-based	Structured communication protocols and pre-case briefings	Distributed situational awareness; extraneous load reduction; collective error detection offsetting primary surgeon's metacognitive failure	Conceptual	Modi et al., 2020 [4]
Team-based	Second attending surgeon as independent cognitive monitor (high-risk or late-day cases)	Independent error detection; crew resource management analog; compensates for depleted surgeon's reduced error-monitoring capacity	Expert consensus	Moscote-Salazar et al., 2023 [2]
Team-based	Empowering team members to raise concerns	Cognitive offloading to experienced nurses and anesthesiologists; collective identification of problems before the depleted surgeon recognizes them	Conceptual	Modi et al., 2020 [4]
Technological	Robotic-assisted surgical systems	Enhanced visualization and motion scaling reduce perceptual and fine-motor cognitive demands; demonstrated mental workload reduction vs. conventional techniques	Prospective controlled	Morse et al., 2025 [13]
Technological	Navigation and augmented-reality guidance	Extraneous cognitive load reduction; real-time spatial cuing offloads working memory demands for anatomical orientation	Prospective controlled	Morse et al., 2025 [13]
Technological	Real-time intraoperative fNIRS/EEG monitoring	Objective biomarker detection of PFC depletion; enables real-time clinical trigger for mandatory breaks or task transfer before performance errors occur	Future direction	Modi et al., 2019 [3]; Modi et al., 2020 [4]
Organizational	Intelligent case sequencing (complex cases scheduled AM)	Aligns highest cognitive-demand cases with peak PFC function and minimal prior accumulation; reduces the case order effect on patient outcomes	Observational	Bagrodia et al., 2012 [6]
Organizational	Fatigue risk management systems (adapted from aviation CRM)	Systemic identification and mitigation of fatigue risk before it manifests as performance error; normalizes institutional acknowledgment of cognitive limits	Conceptual	Laulan et al., 2025 [8]; McCormick et al., 2012 [9]
Organizational	Cultural destigmatization of fatigue acknowledgment	Removes reputational barrier to requesting breaks or declining cases when cognitively depleted; directly addresses surgeon metacognitive failure	Observational	Schlosser et al., 2012 [12]
Organizational	Flush Model-guided scheduling (Laulan framework)	Estimates cumulative cognitive load prospectively; mandates breaks or inserts lower-complexity cases after high-demand procedures to prevent cognitive overflow	Conceptual	Laulan et al., 2025 [8]

Individual-level interventions

At the individual level, scheduled intraoperative and inter-case breaks provide the most direct opportunity for cognitive recovery. Engelmann et al. demonstrated in a randomized controlled trial that intraoperative breaks significantly reduced both mental and somatic fatigue, with measurable improvements in performance metrics; even brief breaks of 10 to 15 minutes can provide meaningful benefit if they involve physical movement, hydration, and mental disengagement from surgical tasks [7]. Nutritional optimization, consuming balanced meals prior to long operating days, avoiding simple sugars that produce rapid glucose fluctuations, and maintaining adequate hydration, supports the metabolic substrate for cognitive function, though practical implementation requires protected meal times and accessible healthy nutrition near surgical areas.

Prioritizing adequate sleep of seven to nine hours per night, maintaining consistent sleep-wake schedules, and employing strategic short naps of 20 to 30 minutes on call days provide foundational cognitive protection, given meta-analytic evidence that even partial chronic sleep restriction meaningfully degrades physician performance. Emerging evidence supports targeted cognitive resilience training: mindfulness-based programs have reduced anxiety and stress responsivity in surgical trainees, and mental rehearsal of surgical procedures has enhanced psychomotor performance while attenuating stress responses [3].

Team-based strategies

Team-based strategies that explicitly distribute cognitive load reduce the burden on the primary surgeon. Structured communication protocols, pre-case briefings, and a culture that empowers team members to speak up with concerns or suggestions enable distributed situational awareness and collective error detection [5]. For procedures of high complexity or scheduled late in the operating day, having a second attending surgeon participate not merely as an assistant but as an independent cognitive monitor represents a team-based safeguard that could be formalized into fatigue risk management protocols, analogous to aviation's two-pilot model, wherein the second crew member provides independent verification of critical decisions and monitors for signs of impaired judgment in the other.

Technological solutions

On the technological front, robotic surgical systems and navigation technologies can reduce specific cognitive demands through enhanced visualization, motion scaling, and automated guidance; Morse et al. demonstrated in a prospective controlled trial that robotic-assisted navigation in spinal surgery significantly reduced surgeons' mental workload compared to conventional fluoroscopic and computed tomographic techniques [13]. Advances in portable fNIRS and wearable electroencephalography may soon enable real-time intraoperative monitoring of PFC activation dynamics, providing objective biomarker triggers for mandatory breaks or task redistribution before performance errors occur [5].

Organizational interventions

At the organizational level, intelligent case sequencing that places complex, high-risk procedures earlier in the day, fatigue risk management systems adapted from aviation CRM, and cultural change initiatives that destigmatize acknowledgment of fatigue collectively address root causes rather than downstream symptoms [5,7]. The Flush Model framework by Laulan et al. provides a practical conceptual tool for surgeons and schedulers to estimate cumulative cognitive load and plan mitigation proactively, such as inserting lower-complexity cases or mandatory breaks after cognitively demanding procedures [8].

Future directions and research gaps

Despite growing recognition of decision fatigue as a patient safety concern, substantial knowledge gaps remain. Real-time neuroimaging during actual surgical procedures represents a critical frontier: advances in portable fNIRS and wearable EEG may soon enable intraoperative monitoring of PFC activation dynamics in authentic operative settings rather than simulated tasks [5]. The identification of reliable biomarkers, whether neural, physiological, or behavioral, would enable both objective research outcome measurement and clinical triggers for real-time mitigation. Novel intraoperative cognitive load monitoring approaches, such as those described in the "surgical sabermetrics" framework, represent promising early steps. The roles of specific neurotransmitter systems in surgical decision fatigue remain largely inferential, and direct pharmacological or neurochemical imaging studies are needed to clarify mechanisms and identify potential intervention targets [10].

The proposed taxonomy of surgical micro-decisions requires empirical validation and refinement through systematic observation across diverse specialties, procedure types, and practice environments. Most proposed mitigation strategies lack rigorous evaluation through randomized controlled trials: high-priority targets include optimal break timing and content, nutritional intervention protocols, cognitive training program design, technology-based cognitive offloading tools, and system-level scheduling algorithms [5,7]. Such trials should incorporate objective performance measures, patient outcome data, and cost-effectiveness analyses. Implementation science approaches are equally needed to understand barriers and facilitators to adoption, since identifying effective interventions is insufficient without strategies for sustainable translation into routine practice.

Finally, the ethical dimensions of decision fatigue, including professional responsibility when fatigued, appropriate disclosure to patients, and organizational obligations, require engagement with bioethics, health policy, and professional societies to establish normative frameworks that balance access to care, surgeon well-being, and patient safety [2].

## Conclusions

Decision fatigue in attending surgeons is a neurobiologically grounded phenomenon with measurable impacts on performance and patient outcomes. The cumulative cognitive burden of hundreds of micro-decisions across an operating day progressively depletes finite neural resources, particularly within the prefrontal cortex, through mechanisms encompassing PFC dysfunction, neurotransmitter dysregulation, and metabolic constraints. This depletion manifests in prolonged operative times, increased error rates, degraded clinical judgment, and elevated complication risks, with a characteristic temporal trajectory moderated by case complexity, inter-case recovery, circadian rhythms, chronic sleep debt, individual resilience, and systemic organizational factors. A critical and clinically consequential feature of this state is that surgeons frequently underestimate their own degree of impairment, underscoring the need for objective monitoring and system-level safeguards rather than sole reliance on individual self-regulation.

Addressing decision fatigue requires a comprehensive, multilevel response encompassing individual strategies such as strategic breaks, nutrition optimization, sleep hygiene, and cognitive resilience training; team-based approaches that distribute cognitive load and leverage collective expertise, including the formalized use of a second attending surgeon as independent cognitive monitor for high-complexity or late-day cases; technological solutions including robotic-assisted systems, navigation platforms, and emerging intraoperative cognitive monitoring tools; and organizational interventions including intelligent case scheduling, fatigue risk management systems, and cultural change that destigmatizes the acknowledgment of cognitive limits. Recognizing decision fatigue as a neurobiological reality rather than a character flaw represents a paradigm shift with profound implications for surgical practice, education, and healthcare system design. Just as surgeons would not operate with uncorrected visual or motor impairment, optimal patient care demands that cognitive depletion be proactively managed rather than silently endured. Sustained investment in research, interdisciplinary collaboration, and collective commitment from clinicians, institutions, and policymakers offer the most promising path toward transforming decision fatigue from an unacknowledged threat into a managed and ultimately mitigated risk, one that is substantially preventable through the systematic implementation of known strategies at the individual, institutional, and policy levels.
